# Characterization of BoHV-5 field strains circulation and report of transient specific subtype of bovine herpesvirus 5 in Argentina

**DOI:** 10.1186/1746-6148-7-8

**Published:** 2011-02-07

**Authors:** Silvina S Maidana, María F Ladelfa, Sandra E Pérez, Patricia M Lomónaco, María P Del Médico Zajac, Anselmo Odeón, Javier Blanco Viera, Gustavo Combessies, Norberto Fondevila, María Palacios, Julien Thiry, Benoît Muylkens, Etienne Thiry, Sonia A Romera

**Affiliations:** 1 Virology Institute, Veterinary and Agricultural Science Research Centre (CICVyA), National Institute of Agricultural Technology (INTA), N. Repetto y Los Reseros S/N, CC25 (B1712WAA), Castelar, Buenos Aires, Argentina; 2 Virology and Viral Diseases, Department of Infectious and Parasitic Diseases, Faculty of Veterinary Medicine, University of Liège, Boulevard de Colonster, 20, B43b, B-4000 Liège, Belgium; 3 Consejo Nacional de Investigaciones Científicas y Tecnológicas (CONICET), Rivadavia 1917 (C1033AAJ), Ciudad Autónoma de Buenos Aires, Argentina; 4 Laboratorio Azul, Av. 25 de Mayo 479/485 (7300), Azul, Buenos Aires, Argentina; 5 National University of the Center of Buenos Aires Province, Faculty of Veterinary Medicine, Department of Animal Health and Preventive Medicine, Virology Area, Paraje Arroyo Seco s/n, Tandil (7000), Buenos Aires, Argentina

## Abstract

**Background:**

Bovine herpesvirus 5 (BoHV-5) is a member of the subfamily *Alphaherpesvirinae *responsible for meningo-encephalitis in young cattle. The first case of bovine meningo-encephalitis associated with a herpesvirus infection was reported in Australia. The current geographical distribution of BoHV-5 infection is mainly restricted to South America, especially Brazil and Argentina. Outbreaks of BoHV-5 are regularly observed in Argentina suggesting the circulation of the virus in the bovine population.

**Results:**

Seventeen field strains of BoHV-5 isolated from 1984 to now were confirmed by differential PCR and subjected to restriction endonuclease analysis (REA). Viral DNA was cleaved with BstEII which allows the differentiation among subtypes a, b and non a, non b. According to the REA with BstEII, only one field strain showed a pattern similar to the Argentinean A663 strain (prototype of BoHV-5b). All other isolates showed a clear pattern similar to the Australian N569 strain (prototype of BoHV-5a) consistent with the subtypes observed in Brazil, the other South-American country where BoHV-5 is known to be prevalent. The genomic region of subtype b responsible for the distinct pattern was determined and amplified by PCR; specifically a point mutation was identified in glycoprotein B gene, on the BstEII restriction site, which generates the profile specific of BoHV-5b.

**Conclusions:**

This is the first report of circulation of BoHV-5a in Argentina as the prevailing subtype. Therefore the circulation of BoHV-5b was restricted to a few years in Argentina, speculating that this subtype was not able to be maintained in the bovine population. The mutation in the gB gene is associated with the difference in the restriction patterns between subtypes "a" and "b".

## Background

Bovine herpesviruses 1 (BoHV-1) and 5 (BoHV-5) are closely related alphaherpesviruses infecting cattle [1,2]. The major difference between BoHV-5 and BoHV-1 is the distinct ability of the former to cause neurological disease in cattle [3,4]. In a natural infection via the respiratory tract, BoHV-1 and BoHV-5 replicate similarly in the nasal mucosa but they differ in their neuroinvasiveness [5,6]. BoHV-1 neuroinvasion usually does not go further than the first order neuron located in the trigeminal ganglia, where the latent infection is established, whereas BoHV-5 is able to infect different regions of the brain [7-9].

The BoHV-5 genome is 137,821 base pairs (bp) long and is approximately 2,000 bp longer than the BoHV-1 genome, with a G + C base composition of 75% [1,6]. These viruses exhibit an average of 82% amino acid identity amongst different proteins. The most similar proteins (≥ 95% amino acid identity) are those involved in viral DNA replication and processing of virion proteins [1,6].

Because of the great similarity between BoHV-1 and BoHV-5, the differentiation by conventional techniques such as viral isolation, immunofluorescence and neutralization test is difficult and has implications for the diagnosis as well as for BoHV-1 vaccination and eradication programs [10,11]. However, these viruses exhibit some important genetic and immunogenic differences, which would explain their pathogenic and epidemiological characteristics [4,12,13]. BoHV-5 is the causal agent of meningo-encephalitis; a condition of low morbidity and high lethality. It usually affects cattle up to 8 months old [14] although older animals can also be affected. This neurological disease is associated with a variety of clinical signs, including tremors, circling, bruxism, incoordination and recumbency, followed by convulsions, padding and inevitable death [15,3,17,5]. Although sporadic cases of meningo-encephalitis by BoHV-5 have been reported in Australia [3,18], Italy [19] and Hungary [20], BoHV-5 infection and disease are more frequent in Argentina and Brazil; where numerous outbreaks have been described in the last decades [16,21-24]. The rare occurrence of BoHV-5 neurological disease in areas where BoHV-1 infection is endemic could be explained by cross-protection induced by BoHV-1 natural infection or vaccination [25,2,27].

Restriction endonuclease analysis (REA) has been widely used to compare BoHV-1 isolates [28,29]. It proved to be particularly useful for the differentiation among various ruminant alphaherpesviruses antigenically related to BoHV-1, which show distinct DNA fingerprints [30,31]. Additionally, this technique allows the subtyping of viruses that apparently belong to the same type [28,32].

Traditionally, the differentiation between BoHV-1 and BoHV-5 is based on the clinico- epidemiological characteristics of the outbreaks followed by REA of viral DNA after virus isolation. However, it is not highly precise because BoHV-1 can also be responsible for neurological disease [33,34]. Furthermore, immunoassays using monoclonal antibodies [4,35,36], PCR followed by REA [37], nested PCR [13] and multiplex PCR [38-40] are also available.

Different strains of BoHV-5 were previously classified and designed as BoHV-5 "a"; BoHV-5 "b" and BoHV-5 "non-a, non-b" according to REA [4,41]. A subtle antigenic difference was shown between subtypes "a" and "b" [4].

The first outbreak of BoHV-5 in Argentina was described in 1982; when the reference strain A663 was isolated. This viral strain was classified by REA as subtype "b" which has only been described in Argentina so far. Therefore, the aims of this study are to compare and characterize by REA the genomes of Argentinean BoHV-5 isolates obtained from cattle with neurological disease since 1984 until now. Furthermore, we identified the mutation that leads to the change in the restriction pattern of the strain A663. We describe a simple diagnostic method based on PCR-REA system that allows an easy discrimination between BoHV-5 "a" and "b" subtypes.

## Results

**Figure 1:**
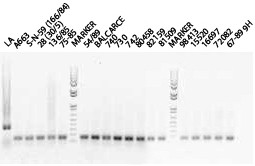
**Ethidium bromide stained 1% agarose gel electrophoresis of the amplified products by multiplex-PCR for BoHV-1 (354 bp) and BoHV-5 (159 bp) glycoprotein C gene**. Marker used was 1 Kb plus marker (Invitrogen)

Most of the isolates included in this study come from outbreaks that occurred between 1982 and 2007 in Buenos Aires province (Argentina). As summarized in Table 1, most of the field BoHV-5 strains were isolated and amplified from the brain of cattle affected with encephalitis. The virus was also isolated from ocular swabs and from the lung of animals with respiratory disease. All viruses isolated from tissues of cattle were successfully amplified. PCR analysis allowed the differentiation between BoHV-1 and BoHV-5 based on the size of the PCR products (159 bp for BoHV-5 and 354 bp for BoHV-1). All field isolates from cattle were BoHV-5 (figure 1).

**Table 1:** Bovine herpesvirus 5 isolates and reference strains grouped in subtypes according to restriction endonuclease analysis

Subtype	Year	Strain	Sample	Origin and Clinical Signs
a	1985	N569	Brain	Australia - Encephalitis (Ref strain)
		28 (30/5)	Lung	Argentina - No data
		136/85	Brain	Argentina -No data
		75/85	Brain	Argentina -No data
	1989	67/89 9H	Ocular Swabs	Balcarce (Buenos Aires, Argentina). Ocular lesions, mouth and prepuce.
		54/89	Lung	Tandil (Buenos Aires, Argentina). Respiratory signs, 10% mortality.
	1999	Balcarce	Brain	Balcarce (Buenos Aires, Argentina). Encephalitis
		740	Brain	Monte (Buenos Aires, Argentina). Encephalitis
		730	Brain	Benito Juarez (Buenos Aires, Argentina). Encephalitis.
		742	Brain	Coronel Suarez (Buenos Aires, Argentina). Encephalitis.
		80458	Brain	Daireux (Buenos Aires, Argentina). Congestion of the brain without malacia.
		82159	Brain	La Plata (Buenos Aires, Argentina). Necrosis in both frontal lobes of the brain.
		81509	Brain	No data. Brain with malacia in both frontal lobes.
	2000	98413	Brain	Guaminí (Buenos Aires, Argentina). Encephalitis.
	2002	15520	Brain	Santa Fe (Argentina). Encephalitis.
		16697	Brain	La Pampa (Argentina). Encephalitis.
	2007	72082	Brain	Monte (Buenos Aires, Argentina). No data.

b	1982	A663	Brain	Argentina - Encephalitis (Ref. strain)
	1984	S-N-59 (166/84)	Brain	Argentina - No data

**Figure 2:**
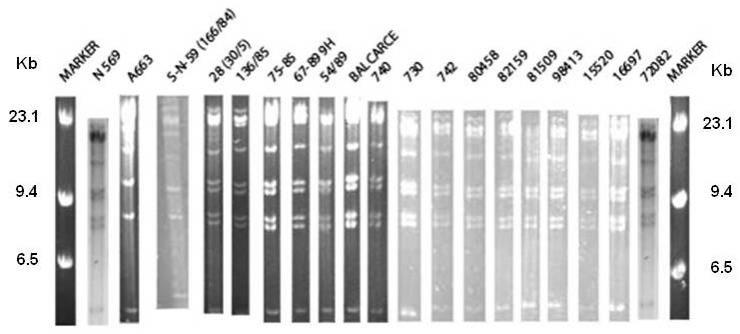
**BstEII restriction endonuclease profiles of the isolates and the reference strains**. Marker: molecular weight marker (lambda Hind III, Invitrogen).

Most field isolates (16 out of 17 isolates) showed a BstEII REA pattern similar to the reference strain N569, prototype of BoHV-5a (Figure 2). Only one field strain (S-N-59 (166/84)) presented a REA pattern similar to the Argentinean reference strain A663 prototype of BoHV-5b. The S-N-59 (166/84) was isolated in 1984, one year after the isolation of the strain A663 (Lane 4, Figure 2).

**Figure 3:**
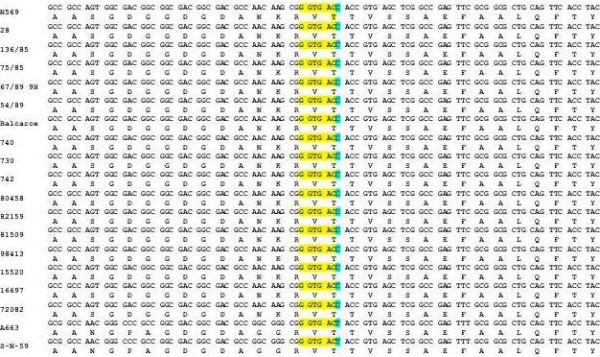
**Localization of the point mutation in the BoHV-5 subtype "b"**. Multiple nucleotide alignment of 87 bp of the UL27 region surrounding the mutation; the restriction site and the mutation are highlighted. Multiple amino-acid alignment of the gB region surrounding the mutation; it is located in the third position (58295 nt position of the genome AY261359) of a threonine codon present in the three strains analyzed, indicating that the mutation does not produce an amino acid change.

A fragment of 534 pb of the UL27 gene, encoding the BoHV-5 glycoprotein B (gB) from the reference strain of BoHV-5 and seventeen isolates of BoHV-5 were sequenced. A consistent alignment provided by a 500 bp fragment from this gene revealed a point mutation (C to T) in the BstEII restriction site of subtype "b" of BoHV-5, responsible for the variation in the REA pattern. The point mutation is shown in alignment with the reference subtypes of BoHV-5 and the isolates (figure 3).

**Figure 4:**
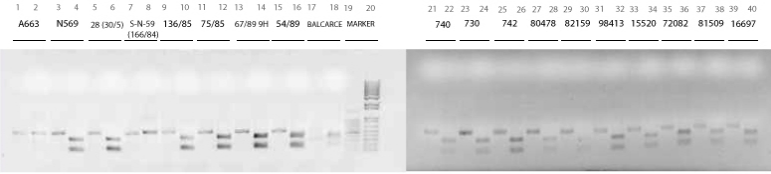
**PCRs amplicons before and after digestion with BstEII of all isolates andreference strains**. Markers used: lane 19) 100 bp DNA Ladder (Promega); lane 20) 1 Kb plus marker (Invitrogen). In gel we added e bar that includes the lines with the undigested PCR product and digested PCR product for each virus. The left line of each virus: the amplicon was digested with the enzyme BstEII.

The specific 534-bp PCR product was consistently obtained as a clear electrophoretic band from all isolates and reference strains of BoHV-5. The digestion of the PCR products with the restriction enzyme BstEII generated distinct restriction patterns depending on the origin of the amplified viral DNA (BoHV-5 "a" or "b" subtypes): 152 and 382 bp for BoHV-5 "a" subtype and 534 bp for BoHV-5 b (Figure 4)

## Discussion

A high seroprevalence for BoHV-1 was reported in Argentina [42]. By now, the proportion of this percentage that can be attributed to BoHV-5 is unknown since diagnostic tests do not differentiate between anti- BoHV-1 and -BoHV-5 antibodies [10,26]. Although the clinical manifestations of BoHV-1 and 5 are slightly different, this is not sufficient to allow a precise distinction between virus species. Furthermore, cases of meningo-encephalitis can also be consequence of BoHV-1 infection [43]. Neighboring countries have reported the circulation of BoHV-5 subtypes "a" and non-"a" non -"b" although Argentina was the first and the only country to report the isolation of a BoHV-5 strain belonging to subtype "b" [41,44].

It has been reported that some outbreaks attributed to the subtype "a" group occurred with respiratory signs in the absence of nervous manifestations (67/89 9H, 54/89). Indeed, in diseased animals, virus infection is not restricted to the central nervous system [21]. Further studies will be required to determine why these BoHV-5 isolates were non-neuropathogenic under these natural conditions.

Several outbreaks occurring between 1981 and 1984, mainly in the central region of Argentina, were determined to be caused by BoHV-5 (9 out of 10 isolates) [21]. In many cases there were no records of morbidity and mortality, except for the outbreak in which the strain A663 was isolated, where 15% (81 out of 540) animals died. Ten percent of the isolates recovered during this period showed slower growth and smaller plaques in tissue culture than the Jura strain of BoHV-1. In this report, we also observed one isolate of BoHV-5 with poor growth in cell culture (1/17), which was classified as subtype "b". This field strain was isolated in 1984 from a meningo-encephalitis outbreak in Arroyo Corto, Saavedra district, located southwest of the Buenos Aires province; the affected herd consisted of 158 bovines; with only one fatality case. This farm had presented a similar outbreak in July-August 1983. Interestingly, this strain affected older animals (18 to 26 months) than the historical case attributed to A663 (5 to 18 months).

According to our study, it is evident that in Argentina both subtypes "a" and "b" circulated for some time and, apparently the circulation of subtype "a" is more recent. As in Brazil, the co-circulation of subtypes "a" and non "a", non "b" was reported [41], further studies are required to determine whether subtype non "a" non "b" is present or will appear in Argentina and whether subtype "b" will definitely disappeared.

In this study, we were also able to determine the region of the subtype "b" genome which is responsible for the different BstEII restriction patterns. The C-T substitution observed in the gene encoding gB, a glycoprotein that is involved in the entry and cell-to-cell spread of virus [45-48] did not introduce an amino-acid change. The digestion of the PCR products with the restriction enzyme BstEII allowed the easy discrimination between "a" and "b" subtypes The diagnostic system based on PCR products has been tested on seventeen isolates and references strains, however before considering the general applicability of this assay it will be necessary to analyze more samples of all BoHV-5 subtypes.

The fragment amplified by PCR shows significant differences at the amino-acid level when compared with the published sequence of this portion of the gene. It will be necessary to conduct further studies to prove whether the changes present in the gB gene of subtype "b" viruses can be related to a specific pattern of virus spread and properties, as observed by Schudel and collaborators [21] regarding the formation of smaller plaques in cell culture and slower growth kinetics with respect to the reference virus.

A further epidemiological study would be required to investigate why the circulation of subtype "b" viruses is restricted to Argentina and why it was only observed in 1982 and 1984. It would also be important to determine whether these isolates exhibit a distinct behavior *in vivo*.

## Conclusion

According to our knowledge, this is the first report on the circulation of BoHV-5 subtype "a" in Argentina. We determined that a mutation in the gB gene is responsible for the difference in the restriction pattern between subtypes "a" and "b". According to the number of samples analyzed, most of the field isolates - including the current ones - belong to subtype "a". Subtype "b" was only isolated during a transient period in 1982 and 1984, and apparently is no longer circulating in the country.

## Methods

### Viruses and cells

BoHV-5 strains were obtained by isolation from samples of naturally infected animals. The virus isolates used in this study are shown in Table 1. They were collected from our and other laboratories across Argentina. All viruses were propagated in Madin Darby Bovine Kidney (MDBK) cells grown in Eagle's minimal essential medium (E-MEM) supplemented with 2% fetal bovine serum (FBS) [49]. The strains N569 and A663 representative of BoHV-5 "a" and BoHV-5 "b" subtypes, respectively, were used as reference viruses [4,41,50,51].

### Viral DNA extraction

Viruses were inoculated in tissue culture flaks (175 cm^2^) with nearly confluent, overnight grown MDBK monolayer, at a multiplicity of infection of 0.1 and incubated at 37°C and 5% CO2. When cytopathic effect reached 80-90% (48 h), the culture medium was clarified by low speed centrifugation and supernatant centrifuged at 100,000 × *g *for 1 h. The viral pellet was re-suspended in buffer TE with 1% NP40 (10 mM Tris; 1mM EDTA; pH 8; 1% NP40) and incubated at 56°C in thermostatic bath. The suspension was ultra-centrifuged in a saccharose gradient at 100,000 × *g*. The viral pellet was re-suspended in TE (10 mM Tris, 1 mM EDTA; pH 8) and treated with 10% sodium dodecyl sulfate (SDS) and 0.1 mg/ml proteinase K for 1 h at 60°C. After digestion, the viral DNA was extracted twice with an equal volume of phenol:chloroform:isoamyl alcohol (25:24:1) and precipitated with 2 volumes of cold ethanol and 0.3 M NaCl. The pellet obtained was rinsed with 600 μl of 70% ethanol, dried and re suspended in 50 μl of nuclease free water and stored at -20°C until use. DNA concentration and purity was measured by BioPhotometer (Eppendorf Scientific, Westbury, NY) at 260 and 280 nm.

### Differential PCR assay

The PCR designed by Claus and collaborators [39] was carried out with some modifications. The primers were B1 specific for BoHV-1 (5-CAA CCG AGA CGG AAA GCT CC-3_nt 185-204); B5 specific for BoHV-5 (5-CGG ACG AGA CGC CCT TGG-3_nt 322-339) and consensus primer designated Bcon (5-AGT GCA CGT ACA GCG GCT CG-3_nt 519-538). The amplification product was 159 bp from BoHV-5 and 354 bp from BoHV-1 (glycoprotein C gene). The multiplex-PCR was prepared in a reaction containing 50 ng of extracted DNA and 12.5 μl of mix-PCR consisting of 0.4 pmol of each primer (B1, B5, and Bcon); 1.5 mMol dNTPs (Invitrogen TM Life Technologies, USA); 2.5 units of *Taq *DNA Polymerase (Invitrogen TM Life Technologies, USA); 1X PCR buffer (20 mM Tris-HCl pH 8.4, 50 mM KCl); 1.5 mM MgCl_2 _and ultrapure sterile water to a final volume of 12.5 μl. Amplification reactions were performed in a thermocycler (Biometra TRIO - Thermoblock) under the following conditions: one step of 10 min at 96°C followed by 25 cycles of 1 min at 96°C; 1 min at 58°C; 1 min at 72°C and one last extension step of 10 min at 72°C. The products were analyzed on 1% agarose gel electrophoresis, stained with ethidium bromide (0.5 μg/ml) in TBE buffer pH 8.4 (89 mM Tris, 89 mM boric acid, 2 mM EDTA) and visualized under UV light.

### Restriction endonuclease analysis (REA)

Four μg of viral DNA from each strain were cleaved with the restriction enzyme BstEII under the conditions recommended by the manufacturer (Promega). The digestion products were separated by electrophoresis on a 0.7% agarose gel at 50 V O.N. using TBE buffer pH 8.4 (89 mM Tris, 89 mM boric acid, 2 mM EDTA). The gels were stained with ethidium bromide (1 μg/μl) and photographed under UV light.

### UL27 PCR assay

Primers were designed based on the published sequence of BoHV-5 (Genbank accession number: AY261359) [1] (UL27F: 5-CTG-GCA-CGA-TCG-AAC-GGC-A-3 nt 58116 and UL27R: 5-AGC-AGC-TCG-TTG-TCC-TCG-C-3 nt 58651). A study conducted *in silico *with bioinformatic tools showed that the site that determines a type "b" pattern is included in the open reading frame of the glycoprotein B (UL27). DNA sequence analysis to locate the discriminative mutant region between BoHV-5a and BoHV-5b within the UL27 target gen was performed using Vector NTI Suite version 8.0 (InforMax Inc-Invitrogen, Merelbeke, Belgium). The assay was performed with purified DNA from strains of different subtypes. Amplification was carried out in a 50 μl reaction mix containing 5 ng of template DNA, *Taq *DNA polymerase buffer (New England Biolabs, NEB, Ipswich, MA, USA), 2 mM MgCl_2_, 6% DMSO, 200 μM dNTPs, 0.3 μM of both forward and reverse primers and 1U TaqDNA polymerase (NEB). Annealing temperatures were optimized for each primer pairs. PCR products were confirmed by electrophoresis on a 2% agarose gel.

### Digestion of amplified product with BstEII (PCR-REA assay)

The purified PCR products were digested with the restriction enzyme BstEII under the conditions recommended by the manufacturer (Promega). The resulting products were separated by electrophoresis in 1% agarose gels and were visualized by ethidium bromide staining under UV light.

### Sequences analysis

Amplified products were purified using Illustra GFX™ PCR, DNA and gel band purification kit (GE Healthcare, Diegem, Belgium). The quality of all DNA preparations was evaluated by agarose gel electrophoresis. Sequencing reactions were performed with BigDye Terminator v3.0 kit (Applied Biosystems, Lennik, Belgium) and analyzed with ABI Prism 3730 DNA Analyzer (Applied Biosystems). Each product was sequenced twice in both directions using forward and reverse primers. The deduced nucleotide and amino-acid sequences were edited; aligned and analyzed with BioEdit version 7.0.5.3 [52].

## Competing interests

The authors declare that they have no competing interests.

## Authors' contributions

SM and SR designed the experiments, analysed the data and drafted the manuscript together. SM performed the experiments. ML, AO, JBV, GC, NF and MD gently surrendered the field isolates. BM, JT and ET participated in the molecular genetic studies and interpretation of data. SP and MP helped to draft the manuscript. FL participated in the amplification of some field isolates. All authors read and approved the final manuscript.
